# To the Editors of the Medical and Physical Journal

**Published:** 1808-01

**Authors:** Henry Le Gros


### *To the Editors of the Medical and Physical Journal.*

GENTLEMEN,

I Beg leave to state a curious case, which has lately fallen under my observation. One of the seamen of his Majesty's ship Barfleur, whose face was deeply marked by the small-pox, in the action of the 22d of July had his face much burnt on the left side by a cartridge, which exploded in his hands. The effect of this was, that the cuticle and reti mucosum, over the whole surface, were destroyed. The parts sloughed off, healed as a common ulcer, and the man at this moment has one side of his face quite smooth, and the other marked as before the accident. I am, &c.

HENRY LE GROS.

